# Candidate Polymorphisms and Severe Malaria in a Malian Population

**DOI:** 10.1371/journal.pone.0043987

**Published:** 2012-09-05

**Authors:** Ousmane Toure, Salimata Konate, Sibiri Sissoko, Amadou Niangaly, Abdoulaye Barry, Abdourahmane H. Sall, Elisabeth Diarra, Belco Poudiougou, Nuno Sepulveda, Susana Campino, Kirk A. Rockett, Taane G. Clark, Mahamadou A. Thera, Ogobara Doumbo, in collaboration with The MalariaGEN Consortium

**Affiliations:** 1 Malaria Research and Training Centre, Faculty of Medicine, Pharmacy and Dentistry, University of Bamako, Bamako, Mali; 2 Centre hospitalier Universitaire Gabriel Toure Hospital, Bamako, Mali; 3 Faculty of Epidemiology and Population Health, London School of Hygiene and Tropical Medicine, London, United Kingdom; 4 Wellcome Trust Sanger Institute, Hinxton, Cambridge, United Kingdom; 5 Wellcome Trust Centre for Human Genetics, University of Oxford, Oxford, United Kingdom; 6 Faculty of Infectious and Tropical Diseases, London School of Hygiene and Tropical Medicine, London, United Kingdom; The George Washington University Medical Center, United States of America

## Abstract

Malaria is a major health burden in sub-Saharan African countries, including Mali. The disease is complex, with multiple genetic determinants influencing the observed variation in response to infection, progression, and severity. We assess the influence of sixty-four candidate loci, including the sickle cell polymorphism (HbS), on severe malaria in a case-control study consisting of over 900 individuals from Bamako, Mali. We confirm the known protective effects of the blood group O and the HbS AS genotype on life-threatening malaria. In addition, our analysis revealed a marginal susceptibility effect for the CD40 ligand (CD40L)+220C allele. The lack of statistical evidence for other candidates may demonstrate the need for large-scale genome-wide association studies in malaria to discover new polymorphisms. It also demonstrates the need for establishing the region-specific repertoire of functional variation in important genes, including the glucose-6-phosphatase deficiency gene, before embarking on focused genotyping.

## Introduction

Malaria is a life-threatening parasitic disease transmitted by mosquitoes. Despite the concerted and renewed efforts to control the disease, it still persists as a major health burden, being responsible for 655,000 deaths in 2010, mainly children in Sub-Sahara Africa [1]. In Mali, there are over 800,000 recorded cases of malaria among its ∼14 million people every year, and it accounts for 17 percent of child deaths [1]. Malaria is a complex disease with many genetic and environmental determinants influencing the observed variation in response to infection, progression and severity. Several factors are important for these different phenotypes observed, such as parasite genetic make-up, and host age, state of immunity and genetic background [2]. It has been estimated that 25% of the total variation in mild and severe malaria in a Kenya cohort was explained by host genes [2]. The different geographic distributions of sickle-cell disease, α thalassemia, glucose-6-phosphatase deficiency (G6PD), ovalocytosis, and the Duffy-negative blood group are examples of the general principle that different populations have evolved different genetic variants to protect against malaria (see [3], for a review). The most striking example is the beta-globin HBB gene, in which three different coding SNPs confer protection against malaria: Glu6Val (HbS), Glu6Lys (HbC), and Glu26Lys (HbE). The HbS allele is common in Africa but rare in Southeast Asia, whereas the opposite is true for the HbE allele. However, a more complex picture emerges at the local level, exemplified by the Dogon people of Mali, who have a much lower frequency of the HbS allele than do most other West African groups and instead have a high frequency of the HbC allele [4]. Striking differences in response to malaria infection have also been observed among ethnic groups who live in the same geographical region. For example, it has been observed that the Fulani of Burkina Faso [5] and of Mali [6] have a significantly lower prevalence of malaria parasitaemia and fewer malaria clinical attacks, when compared to other ethnic groups living in neighbouring villages. In addition to the sickle polymorphism (HbS) [7], G6PD (reviewed in [8]), and ABO blood group [9], [10], a number of candidate polymorphisms have been proposed for the reduced risk of severe malaria. For example, these include genes that are relevant to immunity and inflammation such as the tumour necrosis factor (TNF, MHC class III region, reviewed in [11]), Toll-like receptors (TLR-4,9) [12], CD40 ligand (CD40L) [13], the interferon gamma (IFNG) (reviewed in [14], and the Nitric oxide synthase type 2 (NOS2A) genes (reviewed in [15]. Here we investigate whether a number malaria candidate SNPs, including the HbC, HbS and ABO, are associated with severe malaria. Our study is the first to survey malaria candidate SNPs in a Malian population, and we seek to confirm genetic associations found in other studies. We consider a cohort of over 900 individuals recruited in Bamako, predominantly from the Bambara ethnicity, which is under-represented in other genetic epidemiological studies in Western Africa.

## Methods

### Participants, Materials and Methods

#### Ethics Statement

This study was approved by the Faculty of Medicine, Pharmacy and Dentistry (University of Bamako) Ethics Review Committee. All clinical and biological samples were collected and DNA was genotyped following approval by this committee. Written informed consent was obtained from the next of kin, carers or guardians on the behalf of the minors/children participants involved in this study.

#### Study participants

Patient samples were collected as part of ongoing epidemiological studies of severe malaria at the Centre Hospitalier Universitaire Gabriel Toure, Bamako, Mali (malaria cases 541 (57.9%); healthy controls 393 (42.1%)). They had a median age of ∼3 years, and were predominantly from the Bambara ethnic group (53%) (see Table 1).

**Table 1:** Baseline and clinical characteristics.

	Controls (n = 393)		Cases (n = 541)	
Age* (median, range)	(38.0)	(4.0–178.0)	(36.0)	(2.0–173.0)
Gender - male	195	49.6%	300	55.5%
Bamako residence	140	35.6%	200	37.0%
Ethnicity				
Bambara	219	55.7%	272	50.3%
Malinke	55	14%	83	15.3%
Other	48	12.2%	78	14.4%
Peulh	36	9.2%	56	10.4%
Sarakole	35	8.9%	52	9.6%
Clinical phenotype				
Uncomplicated malaria	-	-	83	15.3%
Any severe malaria	-	-	458	84.7%
Any SMA	-	-	304	66.4%
Any CM	-	-	350	75.1%
Both SMA+CM	-	-	247	53.0%
Any RD	-	-	200	42.9%
Malaria death	-	-	72	15.7%

* in months, SMA = severe malarial anaemia, CM = cerebral malaria, RD = respiratory distress.

#### Phenotypic definition

All cases were children admitted to hospital with evidence of *P. falciparum* on blood film and clinical features of uncomplicated and severe malaria [16], [17]. Subjects were defined as having had cerebral malaria (CM) if their Blantyre coma score was less than or equal to 2 on presentation or early during admission. A second phenotypic subset of severe malarial anaemia (SMA) was defined as those subjects having had a haemoglobin concentration of less than 5 g/dl or a haematocrit less than 15%. Participants with co-existing severe or chronic medical conditions (e.g. bacterial pneumonia, kwashiorkor) unrelated to a severe malarial infection were excluded. Most cases had severe malaria (n = 458, 84.7%), but a minority had uncomplicated conditions (n = 83, 15.3%) (see Table 1). Controls were healthy individuals matched for age, ethnicity and residence to severe malaria cases (Table 1). For the purpose of analysis, we either excluded uncomplicated malaria cases or included them as part of the control group. Because of restrictions in sample size, we do not present an analysis on different ethnic groups or specific sub-clinical phenotypes.

#### Sample preparation and genotyping

Genomic DNA samples underwent whole genome amplification through Primer Extension Pre-amplification (PEP) [18], before genotyping on a Sequenom MassArray genotyping platform [19], [20]. Sixty-four malaria candidate SNPs were genotyped, including: Haemoglobin variants C (HbC, rs33930165) and S (HbS, rs334), plus two SNPs that allow an estimate of the ABO blood group [10]. The rs8176719 derived allele results in a non-functional enzyme, and group O individuals are DD, while non-O Individuals are either II or ID. In addition, rs8176746 is involved in the enzyme's substrate selection and therefore defines either the A or B blood groups. The two Sequenom iPLEX reactions designed also included gender-typing SNPs. The selection of SNPs for genotyping was undertaken by the MalariaGEN Consortium, and were selected by interrogation of the literature and ongoing consortial experiments for evidence of association with severe malaria. Full details of polymorphisms can be found at www.malariagen.net, and a list of SNPs typed can also be found in Tables 2 and S1.

**Table 2:** Single nucleotide polymorphisms.

SNP	MajA	MinA	MAF	Controls	Cases	HWE P	OR	LCL	UCL	P
rs3024500	G	A	0.363	0.367	0.358	0.3800	0.963	0.793	1.170	0.7079
rs1800896	C	T	0.332	0.338	0.327	0.4408	0.951	0.781	1.158	0.6151
rs1800890	T	A	0.185	0.195	0.175	0.0183	0.875	0.689	1.112	0.2743
rs17047660	G	A	0.337	0.331	0.342	0.6354	1.047	0.860	1.275	0.6490
rs17047661	A	G	0.219	0.223	0.216	0.5149	0.959	0.766	1.201	0.7182
rs1803632	C	G	0.456	0.447	0.465	0.4712	1.075	0.892	1.295	0.4492
**rs334**	**A**	**S**	**0.024**	**0.038**	**0.010**	**0.6769**	**0.255**	**0.122**	**0.533**	**0.0003**
rs33930165	G	A	0.054	0.059	0.049	0.1788	0.824	0.540	1.256	0.3669
rs7935564	G	A	0.474	0.479	0.468	0.6407	0.955	0.792	1.152	0.6319
rs542998	T	C	0.411	0.436	0.385	0.2672	0.810	0.669	0.980	0.0304
rs2227507	T	C	0.022	0.022	0.021	0.6289	0.960	0.509	1.811	0.8994
rs1012356	A	T	0.487	0.498	0.475	0.1593	0.912	0.758	1.099	0.3331
rs2227491	T	C	0.312	0.314	0.309	0.1876	0.974	0.797	1.190	0.7972
rs2227485	G	A	0.486	0.497	0.476	0.4513	0.920	0.764	1.109	0.3814
rs229587	C	T	0.286	0.266	0.305	0.3735	1.211	0.981	1.493	0.0742
rs1805015	C	T	0.484	0.488	0.479	0.0791	0.966	0.801	1.164	0.7152
rs2230739	G	A	0.168	0.167	0.169	0.2478	1.014	0.791	1.300	0.9145
rs10775349	G	C	0.163	0.158	0.167	0.9521	1.065	0.828	1.371	0.6229
rs2297518	A	G	0.085	0.081	0.090	0.2139	1.112	0.797	1.551	0.5323
rs1800482	C	G	0.092	0.089	0.096	0.1582	1.077	0.782	1.484	0.6500
rs9282799	T	C	0.067	0.063	0.071	0.0786	1.146	0.791	1.660	0.4722
rs8078340	T	C	0.251	0.257	0.245	0.3906	0.939	0.758	1.164	0.5659
rs1799969	G	A	0.001	0.001	0.001	0.9812	1.035	0.065	16.566	0.9809
rs5498	G	A	0.125	0.124	0.126	0.3716	1.018	0.769	1.348	0.9005
rs373533	T	G	0.375	0.354	0.397	0.0643	1.200	0.989	1.456	0.0647
rs461645	T	C	0.380	0.360	0.399	0.1011	1.180	0.974	1.429	0.0912
rs17561	T	G	0.177	0.178	0.176	0.6004	0.986	0.774	1.257	0.9110
rs1143634	T	C	0.120	0.113	0.126	0.4473	1.133	0.848	1.513	0.3977
rs8386	T	C	0.146	0.145	0.148	0.3342	1.024	0.788	1.331	0.8578
rs1128127	G	A	0.436	0.424	0.448	0.1413	1.102	0.912	1.332	0.3133
rs187084	C	T	0.238	0.243	0.232	0.0059	0.944	0.755	1.182	0.6171
rs6780995	G	A	0.459	0.455	0.463	0.9004	1.032	0.856	1.244	0.7443
rs708567	G	A	0.483	0.462	0.503	0.4465	1.183	0.977	1.431	0.0849
rs4833095	T	C	0.085	0.078	0.093	0.3797	1.220	0.873	1.704	0.2450
rs5743810	C	T	0.002	0.002	0.001	0.9622	0.503	0.046	5.555	0.5748
rs5743809	T	C	0.047	0.041	0.054	0.7487	1.329	0.852	2.073	0.2096
rs2706384	C	A	0.435	0.449	0.422	0.0082	0.895	0.739	1.084	0.2579
rs20541	C	T	0.176	0.173	0.177	0.3304	1.028	0.789	1.341	0.8361
rs2243250	T	C	0.242	0.244	0.240	0.9126	0.979	0.786	1.219	0.8496
rs1801033	A	C	0.445	0.432	0.457	0.9663	1.105	0.916	1.333	0.2951
rs1555498	C	T	0.436	0.426	0.446	0.3849	1.089	0.903	1.313	0.3740
rs2239704	G	T	0.378	0.383	0.372	0.8615	0.951	0.785	1.153	0.6093
rs909253	T	C	0.419	0.411	0.426	0.1203	1.063	0.878	1.287	0.5316
rs1799964	T	C	0.140	0.144	0.136	0.7781	0.942	0.720	1.232	0.6633
rs1800750	G	A	0.014	0.013	0.015	0.7744	1.115	0.506	2.457	0.7876
rs1800629	A	G	0.134	0.137	0.131	0.5698	0.947	0.721	1.243	0.6940
rs361525	G	A	0.037	0.033	0.040	0.4682	1.242	0.758	2.035	0.3892
rs3093662	G	A	0.072	0.067	0.078	0.4503	1.191	0.832	1.706	0.3401
rs2242665	G	A	0.264	0.249	0.280	0.8738	1.175	0.950	1.452	0.1364
rs17140229	C	T	0.393	0.387	0.399	0.7000	1.053	0.869	1.275	0.5988
rs2075820	A	G	0.424	0.430	0.418	0.9496	0.954	0.790	1.152	0.6250
rs3211938	G	T	0.143	0.157	0.129	0.0345	0.793	0.607	1.037	0.0900
hCD36_G1439C	C	G	0.055	0.060	0.050	0.7433	1.216	0.805	1.836	0.3520
rs4986790	G	A	0.110	0.120	0.100	0.0436	0.819	0.608	1.104	0.1893
**rs8176746**	**A**	**C**	**0.244**	**0.213**	**0.276**	**0.0093**	**1.412**	**1.135**	**1.757**	**0.0020**
**rs8176719**	**I**	**D**	**0.403**	**0.364**	**0.442**	**0.8022**	**1.384**	**1.145**	**1.673**	**0.0008**
**ABO**	**Non-O**	**O**	**0.356**	**0.438**	**0.295**	**NA**	**0.539**	**0.438**	**0.662**	**<0.0001**
rs3092945 F	T	C	0.334	0.387	0.285	0.3588	0.631	0.465	0.856	0.0031
rs3092945 M	T	C	0.346	0.320	0.365	NA	1.219	0.913	1.627	0.1795
rs3092945 Ov	T	C	0.338	0.352	0.327	NA	0.893	0.724	1.101	0.2891
rs1126535 F	T	C	0.128	0.095	0.158	0.5737	1.798	1.157	2.796	0.0092
rs1126535 M	T	C	0.098	0.077	0.113	NA	1.534	0.954	2.468	0.0776
**rs1126535 Ov**	**T**	**C**	**0.112**	**0.085**	**0.134**	**NA**	**1.671**	**1.209**	**2.309**	**0.0019**
rs1050829 F	A	G	0.423	0.401	0.443	0.9805	1.188	0.890	1.585	0.2421
rs1050829 M	A	G	0.418	0.425	0.413	NA	0.954	0.724	1.259	0.7412
rs1050829 Ov	A	G	0.421	0.413	0.427	NA	1.060	0.868	1.294	0.5673
rs1050828 F	G	A	0.162	0.146	0.178	0.9615	1.267	0.862	1.861	0.2281
rs1050828 M	G	A	0.183	0.162	0.198	NA	1.278	0.896	1.824	0.1757
rs1050828 Ov	G	A	0.173	0.154	0.189	NA	1.273	0.981	1.653	0.0699

MinA = minor allele, MajA = major allele, MAF = overall minor allele frequency, HWE P is the Hardy-Weinberg p-value, OR = odds ratio, 95% Confidence interval (LCL, UCL), P = P-value; rs8176746 and rs8176719 are used to infer ABO blood groups; for X chromosome SNPs (rs3092945 (CD40), rs1126535 (CD40), rs1050829 (G6PD-376), rs1050828 (G6PD-202/A-), analyses are presented for separately for females (F) and males (M) and pooled to obtain overall results (Ov), NA not applicable; rs33950507, rs5743611, rs2814778, rs2227478, rs2535611 and rs1801274 did not pass quality control filters and are not presented.

#### Statistical analysis

Genotypic deviations from Hardy-Weinberg equilibrium (HWE) were assessed using a chi-square statistical test. SNPs were excluded from analysis if they had at least 10% of genotype calls missing or there was significant deviation from HWE (p<0.001). Case-control association analysis using SNP alleles/genotypes was undertaken by logistic regression and included the covariates: ethnic group and the HbS polymorphism. In this approach we modelled the SNP of interest assuming several related genotypic mechanisms (additive, dominant, recessive, heterozygous advantage and general models) and reported the minimum p-value from these correlated tests. All analyses were performed using the R statistical package (http://www.r-project.org). Performing multiple statistical tests leads to inflation in the occurrence of false positives and using a permutation approach that accounted for correlation between markers and tests, we estimated a p-value cut-off of 0.002 to be statistically significant. Allele frequency differences were estimated using the *Fst* metric, with values potentially varying from zero (no population differentiation) to one (complete differentiation) [22].

## Results

**Figure 1:**
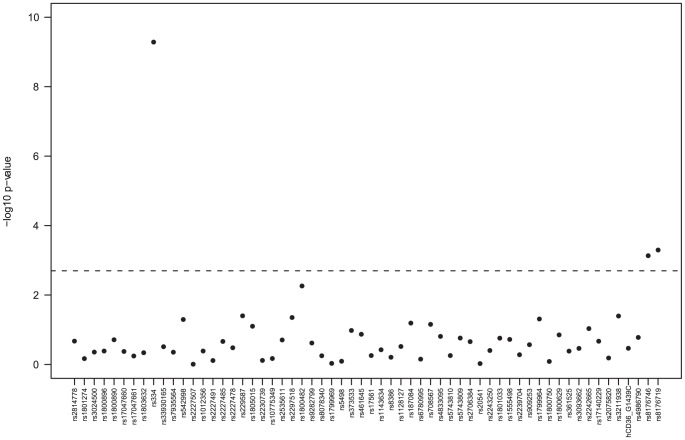
Minimum p-values from tests of association for the autosomal SNPs ^*^. ^*^Genotypic tests of dominant, recessive, general, heterozygous advantage, and additive models, adjusted for HbS and ethnicity; in this analysis controls include uncomplicated malaria cases; the dashed line represents a p-value of 0.002.

Six SNPs were removed from the analysis because they were monomorphic (rs33950507, rs5743611, rs2814778), deviated from HWE in controls (rs2227478, rs2535611) or had high rates of missing genotype calls (rs1801274). Allelic-based tests revealed potential associations of HbS polymorphism (rs334, HBB gene), the O blood group (and its components rs8176746 and rs8176719), and rs1126535 (CD40L+220) with severe malaria (P<0.002) (Table 2). Figure 1 shows the minimum p-values from the genotypic tests applied to the autosomal SNPs, and confirms that the sickle cell (HbS) and ABO polymorphisms (rs8176746, rs8176719) are significantly associated with severe malaria. This genotypic analysis adjusting for the potentially confounding effects of ethnicity (and where appropriate HbS) supports the heterozygous advantage effect of the HbS-AS genotype (Odds ratio AS vs. AA: 0.03, P<6e-10), and the reduced risk in the O blood group (Odds ratio O vs. A/B/AB: 0.58, P = 0.0003) (Table 3). An increased risk from the CD40L+220-C allele was observed for males (Odds ratio C vs. A 2.12, P = 0.05) and females (Odds ratio additive C model 1.67, P = 0.03), and the pooled result was marginally non-significant (Odds ratio additive C model 1.79, P = 0.0045) (Table 3). The G6PD-202 polymorphism has been used as a molecular surrogate for the A- deficiency (see [8], [21] for a review). We found no strong evidence of a G6PD association with severe malaria risk in males, females or overall (Tables 2 and 3, P>0.06); the direction of the odds ratios suggests those with the 202-A (A-) allele were at increased (rather than a decreased) risk of the disease. All our results were insensitive to the inclusion or exclusion of uncomplicated malaria cases in the control group. There were no major allele frequency differences between the Bambara and other ethnic groups (median Fst 0.001, only 3 Fst values greater than 0.01, maximum 0.015).

**Table 3:** Odds ratios* for HbS, ABO, CD40 and G6PD-202.

Polymorphism	Contrast	OR	LCL	UCL	P
HbS (rs334)	AS vs. AA	0.028	0.004	0.209	<6E-10
Blood Group (rs8176719)	O vs. A/B/AB	0.575	0.425	0.777	0.0003
CD40 (rs1126535) – Female	Additive C	1.674	1.045	2.683	0.03
CD40 (rs1126535) – Male	Additive C	2.119	0.990	4.537	0.05
CD40 (rs1126535) – Overall	Additive C	1.787	1.197	2.669	0.0045
G6PD (rs1050828, 202) – Female	AG vs. GG	1.187	0.747	1.887	0.467
G6PD (rs1050828, 202) – Female	AA vs. GG	1.557	0.427	5.684	0.502
G6PD (rs1050828, 202) – Male	A vs. G	1.205	0.718	2.022	0.480

* adjusted for HbS and ethnicity, OR = odds ratio, 95% Confidence interval (LCL, UCL), P = P-value, in this analysis controls include uncomplicated malaria cases.

## Discussion

In our study, we set out to investigate the role of candidate malaria polymorphisms on severe malaria risk in a predominantly Bambara population in and around Bamako. To minimise errors we standardised procedures using case report forms, pre-defined definitions of severe malaria and sub-clinical phenotypes, and ensured all samples underwent genotyping on the same Sequenom MassArray platform with resulting low rates of missing data (<5%). In addition, all analyses were adjusted for ethnicity, minimising the confounding effects and potential false positives arising from population stratification.

Data analysis confirmed the known (∼90%) reduced severe malaria risk from the sickle cell AS genotype [7]. The low frequency of the S allele in the controls (∼3.8%) is in keeping with other populations (see http://www.map.ox.ac.uk/) in West (Burkina Faso 5.2%, Cameroon 6.5%, Gambia 7.6%, Ghana 6.5%) and East (Kenya 6.4%, Malawi 2.7%, Tanzania 7.8%) Africa. The frequency of the HbC allele was 5% in cases and 6% in controls, both greater than the HbS allele, but there was no strong evidence of association (P>0.3). The higher frequency of the HbC allele has been observed in other West African populations [4].

Our analysis also confirms the known protective effects of the blood group O on life threatening malaria, which is thought to act on malaria pathogenesis through the mechanism of reduced *P. falciparum* rosetting [9], [10]. An insight into an underlying molecular mechanism could lead to the development of a new anti-malarial therapy. Our inability to detect associations found in other studies may be due to previously reported false-positives, and methodological issues such as variation in phenotype definition, choice of controls, village surveys versus hospital-based studies, possible heterogeneity in the parasite population, immune status of subjects, and study location. There may also be issues with sample size (statistical power). At the present sample size, we have 90% §power to detect a dominant odds ratio effect of 1.5 with a minor allele frequency of 0.20 and type I error of 5%. Reducing the odds ratio to 1.4 or MAF to 0.13 would lead to 80% power. It is clear that much larger studies are required to detect more modest effects, especially if relying on markers in LD with a causal untyped polymorphism. In this setting, dense SNP genome-wide association strategies are required to discover new candidate genes.

Our analysis revealed a marginal susceptibility effect for the CD40 ligand (CD40L)+220C allele. CD40L is a glycoprotein involved in B cell proliferation, antigen presenting cell activation, and Ig class switching, and therefore important in the immune response to infection [13]. Previous work in the Gambia found the CD40L+220C allele had a non-significant susceptibility effect, and instead identified the genotyped CD40L–726 polymorphism with a significant reduction in risk for severe malaria in males-hemizygous [13]. Similarly, we expected to see a reduced malaria risk with G6PD-202A (A- deficiency), as shown in another Malian population [8] with similar allele frequencies in controls to our study. These discrepancies in CD40L and G6PD results may be due to allelic heterogeneity. For example, it has been shown that the G6PD-202 may not be a good marker of A- deficiency, and other polymorphisms are required to confirm the protective effect [21]. In addition, discrepancies may arise due to differences in linkage disequilibrium (LD) patterns between populations. For example, it is known there are at least five classical haplotypes surrounding the sickle polymorphism in the HBB gene, and the resulting differences in LD can make it difficult to localise HbS using indirect associations [7]. In addition, some functional polymorphisms may be distal from candidate genes and polymorphisms genotyped. For example, it has been suggested that the causal polymorphisms regulating TNF and LTA response may be some distance from the genes [11], [23].

In conclusion, our work reinforces in a Malian (mainly Bambara) population the importance of the sickle cell polymorphism and ABO blood group on severe malaria susceptibility. It may also demonstrate the need for establishing the region-specific repertoire of functional variation in important genes such as G6PD, before embarking on focused genotyping. Proposed large-scale genomewide association studies and resequencing of important genes in a number of Malian (and other African) populations is likely to generate insights into how *P. falciparum* has shaped our genome. These approaches could expose new candidate protective polymorphisms, resulting in functional work to understand underlying molecular mechanisms, leading to the development of therapies and vaccines for malaria control.

## Supporting Information

Table S1 List of all candidate polymorphisms considered in our work.
(DOC)

## References

[1] World Health Organisation (2011) World Malaria Report 2011. Available: http://who.int/malaria/world_malaria_report_2011/en/index.html. Accessed 2012 Aug 14.

[2] Mackinnon MJ, Mwangi TW, Snow RW, Marsh K, Williams TN (2005) Heritability of malaria in Africa. PLoS Med 2: e340. 16259530 10.1371/journal.pmed.0020340 PMC1277928

[3] Campino S, Kwiatkowski D, Dessein A (2006) Mendelian and complex genetics of susceptibility and resistance to parasitic infections. Semin Immunol 18: 411–22. 17023176 10.1016/j.smim.2006.07.011

[4] Agarwal A, Guindo A, Cissoko Y, Taylor JG, Coulibaly D, et al (2000) Hemoglobin C associated with protection from severe malaria in the Dogon of Mali, a West African population with a low prevalence of hemoglobin S. Blood 96: 2358–63. 11001883

[5] Modiano D, Petrarca V, Sirima BS, Bosman A, Nebie I, et al (1995) Plasmodium falciparum malaria in sympatric ethnic groups of Burkina Faso, west Africa. Parassitologia 37: 255–9. 8778668

[6] Dolo A, Modiano D, Maiga B, Daou M, Dolo G, et al (2005) Difference in susceptibility to malaria between two sympatric ethnic groups in Mali. Am J Trop Med Hyg 72: 243–8. 15772314

[7] Jallow M, Teo YY, Small KS, Rockett KA, Deloukas P, et al (2009) Genome-wide and fine-resolution association analysis of malaria in West Africa. Nat Genet 41(6): 657–665. 19465909 10.1038/ng.388 PMC2889040

[8] Guindo A, Fairhurst RM, Doumbo OK, Wellems TE, Diallo DA (2007) X-linked G6PD deficiency protects hemizygous males but not heterozygous females against severe malaria. PLoS Med 4: e66. 17355169 10.1371/journal.pmed.0040066 PMC1820604

[9] Rowe JA, Handel IG, Thera MA, Deans AM, Lyke KE, et al (2007) Blood group O protects against severe *Plasmodium falciparum* malaria through the mechanism of reduced rosetting. . Proc Natl Acad Sci U S A 104: 17471–17476. 17959777 10.1073/pnas.0705390104 PMC2077280

[10] Fry AE, Griffiths MJ, Auburn S, Diakite M, Forton JT, et al (2008) Common variation in the ABO glycosyltransferase is associated with susceptibility to severe Plasmodium falciparum malaria. Hum Mol Genet 17: 567–76. 18003641 10.1093/hmg/ddm331 PMC2657867

[11] Clark TG, Diakite M, Auburn S, Campino S, Fry AE (2009) Tumor necrosis factor and lymphotoxin-alpha polymorphisms and severe malaria in African populations. J Infect Dis 199: 569–75. 19281305 10.1086/596320 PMC2742199

[12] Mockenhaupt FP, Hamann L, von Gaertner C, Bedu-Addo G, von Kleinsorgen C (2006) Common polymorphisms of toll-like receptors 4 and 9 are associated with the clinical manifestation of malaria during pregnancy. J Infect Dis 194: 184–8. 16779724 10.1086/505152

[13] Sabeti P, Usen S, Farhadian S, Jallow M, Doherty T, et al (2002) CD40L association with protection from severe malaria. Genes Immun 5: 286–291. 10.1038/sj.gene.6363877 12140747

[14] Stevenson MM, Riley EM (2004) Innate immunity to malaria. Nat Rev Immunol 4: 169–80. 15039754 10.1038/nri1311

[15] Clark IA, Rockett KA (1996) Nitric oxide and parasitic disease. Adv Parasitol 37: 1–56. 8881597 10.1016/s0065-308x(08)60218-3

[16] Marsh K, Forster D, Waruiru C, Mwangi I, Winstanley M, et al (1995) Indicators of life-threatening malaria in African children. N Engl J Med 332: 1399–404. 7723795 10.1056/NEJM199505253322102

[17] World Health Organisation (1990) Severe and complicated malaria. World Health Organization, Division of Control of Tropical Diseases. Trans R Soc Trop Med Hyg 84 Suppl 2 1–65. 2219249

[18] Zhang L, Cui X, Schmitt K, Hubert R, Navidi W, et al (1992) Whole genome amplification from a single cell: implications for genetic analysis. Proc Natl Acad Sci U S A 89: 5847–51. 1631067 10.1073/pnas.89.13.5847 PMC49394

[19] Wilson JN, Rockett K, Jallow M, Pinder M, Sisay-Joof F, et al (2005) Analysis of IL10 haplotypic associations with severe malaria. Genes Immun 6: 462–6. 15933743 10.1038/sj.gene.6364227

[20] Ross P, Hall L, Smirnov I, Haff L (1998) High level multiplex genotyping by MALDI-TOF mass spectrometry. Nat Biotechnol 16: 1347–51. 9853617 10.1038/4328

[21] Clark TG, Fry A, Auburn S, Campino S, Diakite M, et al (2009) G6PD deficiency and severe malaria: unrecognized allelic heterogeneity is confounding association studies in Africa. Eur J Human Genetics 17: 1080–5. 19223928 10.1038/ejhg.2009.8 PMC2986558

[22] Weir BS, Cockerham CC (1984) Estimating F-statistics for the analysis of population structure. Evolution 38 (6) 1358–1370. 28563791 10.1111/j.1558-5646.1984.tb05657.x

[23] Diakite M, Clark TG, Auburn S, Campino S, Fry AE, et al (2009) A genetic association study in the Gambia using tagging polymorphisms in the major histocompatibility complex (MHC) class III region implicates a BAT2 polymorphism in severe malaria susceptibility. Human Genetics 125: 105–9. 19039607 10.1007/s00439-008-0597-2 PMC2992315

