# Supplementary material for: Candidate Polymorphisms and Severe Malaria in a Malian Population
Source: PLoS One. 2012 Sep 5;7(9):e43987. doi: 10.1371/journal.pone.0043987 (PMC3434208; doi:10.1371/journal.pone.0043987)
Supplement: Table S1 — List of all candidate polymorphisms considered in our work. (DOC) [file pone.0043987.s001.doc]

**Table S1**

**List of all candidate polymorphisms considered in our work**

| Rs number* | SNP Name | Chr | Position | Gene | Ref. allele | Derived allele |
| --- | --- | --- | --- | --- | --- | --- |
| rs1803632 | rs7537937 | 1 | 89582690 | GBP7 | G | C |
| rs2814778 | rs2814778 | 1 | 159174683 | DARC | A | G |
| rs1801274 | rs1801274 | 1 | 159746359 | FCGR2A | T | T |
| rs3024500 | IL10_232424450 | 1 | 206940831 | IL10 | G | A |
| rs1800896 | IL10-1082 | 1 | 206946897 | IL10 | T | C |
| rs1800890 | IL10-3533 | 1 | 206949365 | IL10 | A | T |
| rs17047660 | rs17047660 | 1 | 207782856 | CR1 | A | G |
| rs17047661 | rs17047661 | 1 | 207782889 | CR1 | A | G |
| rs17561 | rs17411697 | 2 | 113537223 | IL1A | G | T |
| rs1143634 | rs1143634 | 2 | 113590390 | IL1B | C | T |
| rs708567 | rs708567 | 3 | 9960070 | IL17RE | G | A |
| rs187084 | TLR9_rs187084 | 3 | 52261031 | TLR9 | C | T |
| rs6780995 | rs6780995 | 3 | 57138419 | IL17RD | G | A |
| rs4833095 | TLR1_rs4833095 | 4 | 38799710 | TLR1 | C | T |
| rs5743611 | TLR1_rs5743611 | 4 | 38800214 | TLR1 | G | C |
| rs5743810 | TLR6_rs5743810 | 4 | 38830350 | TLR6 | C | T |
| rs5743809 | TLR6_rs5743809 | 4 | 38830514 | TLR6 | T | C |
| rs1801033 | rs1801033 | 5 | 41199959 | C6 | A | C |
| rs2706384 | 5-131349023 | 5 | 131826880 | IRF1 | C | A |
| rs20541 | IL13_46457 | 5 | 131995964 | IL13 | C | T |
| rs2243250 | IL4-589 | 5 | 132009154 | IL4 | C | T |
| rs2239704 | LTA 80 | 6 | 31540141 | LTA | G | T |
| rs909253 | LT-alpha_NcoI | 6 | 31540313 | LTA | T | C |
| rs1799964 | TNFa-1031 | 6 | 31542308 | TNF | T | C |
| rs1800750 | TNF-376 | 6 | 31542963 | TNF | G | A |
| rs1800629 | TNF-308 | 6 | 31543031 | TNF | G | A |
| rs361525 | TNF-238 | 6 | 31543101 | TNF | G | A |
| rs3093662 | TNF+851 | 6 | 31544189 | TNF | A | G |
| rs2242665 | rs2242665 | 6 | 31839309 | CTL4 | G | A |
| rs1555498 | rs1555498 | 6 | 137325847 | IL20RA | C | T |
| rs2075820 | rs2075820 | 7 | 30492237 | NOD1 | G | A |
| rs3211938 | CD36_T1264G | 7 | 80300449 | CD36 | T | G |
| CD36_G1439C | CD36_G1439C | 7 | 80302110 | CD36 | G | C |
| rs17140229 | rs17140229 | 7 | 117230283 | CFTR | T | C |
| rs4986790 | rs4986790 | 9 | 120475302 | TLR4 | A | G |
| rs8176746 | rs8176746 | 9 | 136131322 | ABO | C | A |
| rs8176719 | rs8176719 | 9 | 136132719 | ABO | I | D |
| rs33950507 | rs33950507 | 11 | 5248173 | HBB | G | A |
| rs334 | HbS | 11 | 5248232 | HBB | A | T |
| rs33930165 | HbC | 11 | 5248409 | HBB | G | A |
| rs7935564 | rs7935564 | 11 | 5718517 | TRIM5 | G | A |
| rs542998 | rs542998 | 11 | 63487386 | RTN3 | T | C |
| rs2227507 | 12-72245636 | 12 | 68642647 | IL22 | T | C |
| rs1012356 | 12-72247607 | 12 | 68644618 | IL22 | A | T |
| rs2227491 | 12-72249510 | 12 | 68646521 | IL22 | T | C |
| rs2227485 | 12-72250702 | 12 | 68647713 | IL22 | G | A |
| rs2227478 | 12-72251611 | 12 | 68648622 | IL22 | G | A |
| rs229587 | rs229587 | 14 | 65263300 | SPTB | T | C |
| rs2230739 | rs2230739 | 16 | 4033436 | ADCY9 | A | G |
| rs10775349 | rs10775349 | 16 | 4079823 | ADCY9 | G | C |
| rs1805015 | IL4R-63011 | 16 | 27374180 | IL4R | T | C |
| rs2535611 | rs2535611 | 17 | 15861332 | ADORA2B | C | T |
| rs2297518 | rs2297518 | 17 | 26096597 | NOS2 | G | A |
| rs1800482 | hNOS2-954 | 17 | 26128509 | NOS2 | G | C |
| rs9282799 | rs9282799 | 17 | 26128728 | NOS2 | C | T |
| rs8078340 | hNOS2-1659 | 17 | 26129212 | NOS2 | C | T |
| rs373533 | rs373533 | 19 | 6919624 | EMR1 | G | T |
| rs461645 | rs461645 | 19 | 6919753 | EMR1 | T | C |
| rs1799969 | ICAM-1codon241 | 19 | 10394792 | ICAM1 | G | A |
| rs5498 | ICAM-1codon469 | 19 | 10395683 | ICAM1 | A | G |
| rs8386 | GNAS_8386 | 20 | 57485812 | GNAS | C | T |
| rs1128127 | rs3177244 | 22 | 24179132 | DERL3 | G | A |
| rs3092945 | rs3092945 | X | 135729609 | CD40LG | T | C |
| rs1126535 | rs17424229 | X | 135730555 | CD40LG | T | C |
| rs1050829 | G6PD+376 | X | 153763492 | G6PD | T | C |
| rs1050828 | G6PD+202 | X | 153764217 | G6PD | C | T |

Ref. reference, * genome build dbSNP132/GRch37, ensembl version 61
